# Protein malnutrition potentiates the amplifying pathway of insulin secretion in adult obese mice

**DOI:** 10.1038/srep33464

**Published:** 2016-09-16

**Authors:** Nayara Carvalho Leite, Flávia de Paula, Patrícia Cristine Borck, Jean Franciesco Vettorazzi, Renato Chaves Souto Branco, Camila Lubaczeuski, Antonio Carlos Boschero, Claudio Cesar Zoppi, Everardo Magalhães Carneiro

**Affiliations:** 1 Department of Structural and Functional Biology, Institute of Biology, University of Campinas (UNICAMP), Campinas, SP, CEP: 13083-865, Brazil

## Abstract

Pancreatic beta cell (β) dysfunction is an outcome of malnutrition. We assessed the role of the amplifying pathway (AMP PATH) in β cells in malnourished obese mice. C57Bl-6 mice were fed a control (C) or a low-protein diet (R). The groups were then fed a high-fat diet (CH and RH). AMP PATH contribution to insulin secretion was assessed upon incubating islets with diazoxide and KCl. CH and RH displayed increased glucose intolerance, insulin resistance and glucose-stimulated insulin secretion. Only RH showed a higher contribution of the AMP PATH. The mitochondrial membrane potential of RH was decreased, and ATP flux was unaltered. In RH islets, glutamate dehydrogenase (GDH) protein content and activity increased, and the AMP PATH contribution was reestablished when GDH was blunted. Thus, protein malnutrition induces mitochondrial dysfunction in β cells, leading to an increased contribution of the AMP PATH to insulin secretion through the enhancement of GDH content and activity.

Obesity is a worldwide trend that is associated with the development of several co-morbidities such as metabolic syndrome and type 2 diabetes (T2D)^1^. Islet dysfunction appears to be the most important component for T2D development. Generally, this process starts with obesity-induced insulin resistance, and when the β cells of susceptible individuals fail to supply adequate amounts of insulin, hyperglycemia appears and T2D takes place^2^. Insulin resistance leads to an enhancement of glucose-stimulated insulin secretion (GSIS). One of the earliest events reported during this pre-diabetes condition is the deterioration of the first phase and the pulsatile characteristic of insulin secretion^3^,^4^, whereas the second phase seems to be maintained until near the end-phase of diabetes^5^.

The biphasic profile of GSIS reflects the interaction of several controlling pathways. The triggering GSIS pathway is thought to signal the first phase of insulin secretion through the canonical K^+^ATP sensitive channel (K^+^_ATP_)-dependent pathway, whereas the maintenance of the second phase is due to the amplifying or K^+^_ATP_-independent pathways^6^,^7^. The amplifying pathways (AMP PATH) were determined in β cells lacking K^+^_ATP_ function or content, which were still able to secrete insulin in response to an increase in glucose^8^. Mitochondrial metabolism has been proposed to be one of the amplifying mechanisms^9^,^10^. Thus, mitochondria seem to be crucial for the control of insulin secretion, providing ATP for the triggering pathway and other metabolic coupling factors to the AMP PATH.

Protein malnutrition during early life programs metabolism to favor obesity and T2D development during adulthood^11^. Evidence of impaired β cell function in malnourished rodents^12^,^13^, including the impairment of first phase insulin secretion^14^, was demonstrated. Mitochondrial dysfunction due to protein malnutrition points to the emerging role of this organelle as the main site of pancreatic β cell programming^15^.

Nutritional improvement reached by developing countries gave rise to a double burden of malnutrition and overnutrition^16^. Under this situation, nutritional transition can occur so rapidly that individuals may face a deficiency of several nutrients in association with excessive caloric intake^17^. Low-protein and high-fat diets lead to the development of obesity and T2D, and when this occurs in the same subject, undesirable events may be potentiated. An impaired GSIS triggering mechanism, such as altered protein content of the K^+^_ATP_ channel subunits, was reported in obese malnourished mice^18^; however, the impact of obesity and malnourishment on mitochondrial function in β cells and the AMP PATH of insulin secretion is still poorly understood.

Considering the hypothesis that β cell dysfunction is at the center of malnutrition-induced T2D development^19^,^20^ and mitochondria are the main intracellular targets of this programming, investigating the molecular mechanisms by which protein malnutrition induces a metabolic program might provide clues to the establishment of therapeutic targets to avoid the undesirable outcomes. Thus, we investigated the effects of protein malnutrition on the AMP PATH of GSIS in obese mice and the underlying molecular mechanisms.

## Results

### Malnourished obese mice present an increased contribution of the AMP PATH of insulin secretion

**Figure 1:**
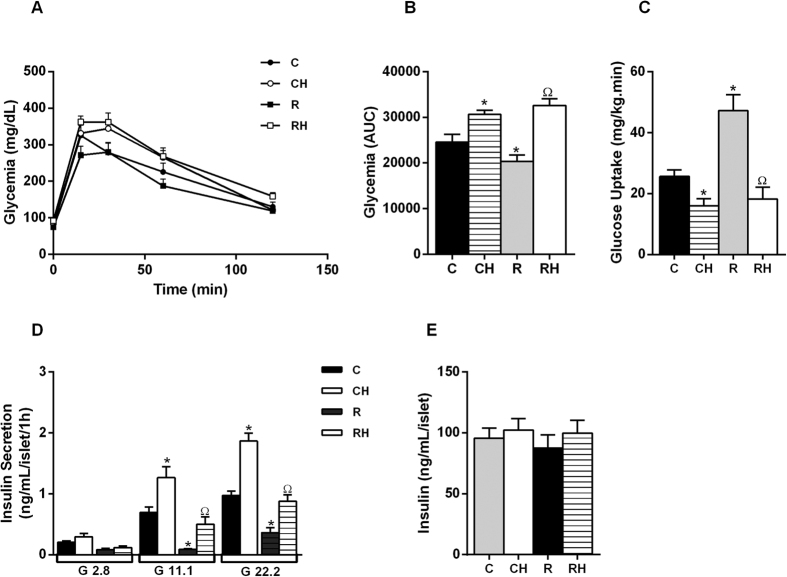
Malnourished obese mice present glucose intolerance, insulin resistance and increase of GIIS: Glucose tolerance test (**A**). The corresponding area under the curve (AUC) is shown in panel (**B**). Glucose Uptake during hyperinsulinemic-euglycemic clamp (**C**). Insulin secretion in response to glucose (**D**). 8–10 observations from 3 independent experiments were made for each situation (n = 4–6 mice per group). Total insulin content (**E**) (p < 0.05) *C; ^Ω^R, in Student’s t test.

Restricted mice displayed decreased body weight (BW) (Fig. S1A, Table S1), increased glucose tolerance, and insulin sensitivity (Fig. 1A–C) with a decrease in plasma insulin levels (Table S1). HFD intake increased BW, fat depots, the Lee index, glucose intolerance, and insulin resistance (Fig. 1A–C) in the CH and RH groups (Table S1). Both of the groups displayed increased total fasting plasma insulin levels (Table S1). The RH group presented diminished energy expenditure. R secreted less insulin in the presence of 22.2 mmol/L glucose compared with C, whereas the mice treated with a HFD displayed higher insulin secretion (Fig. 1D), despite no reported changes in the total content of insulin in islets from all groups (Fig. 1E).

**Figure 2:**
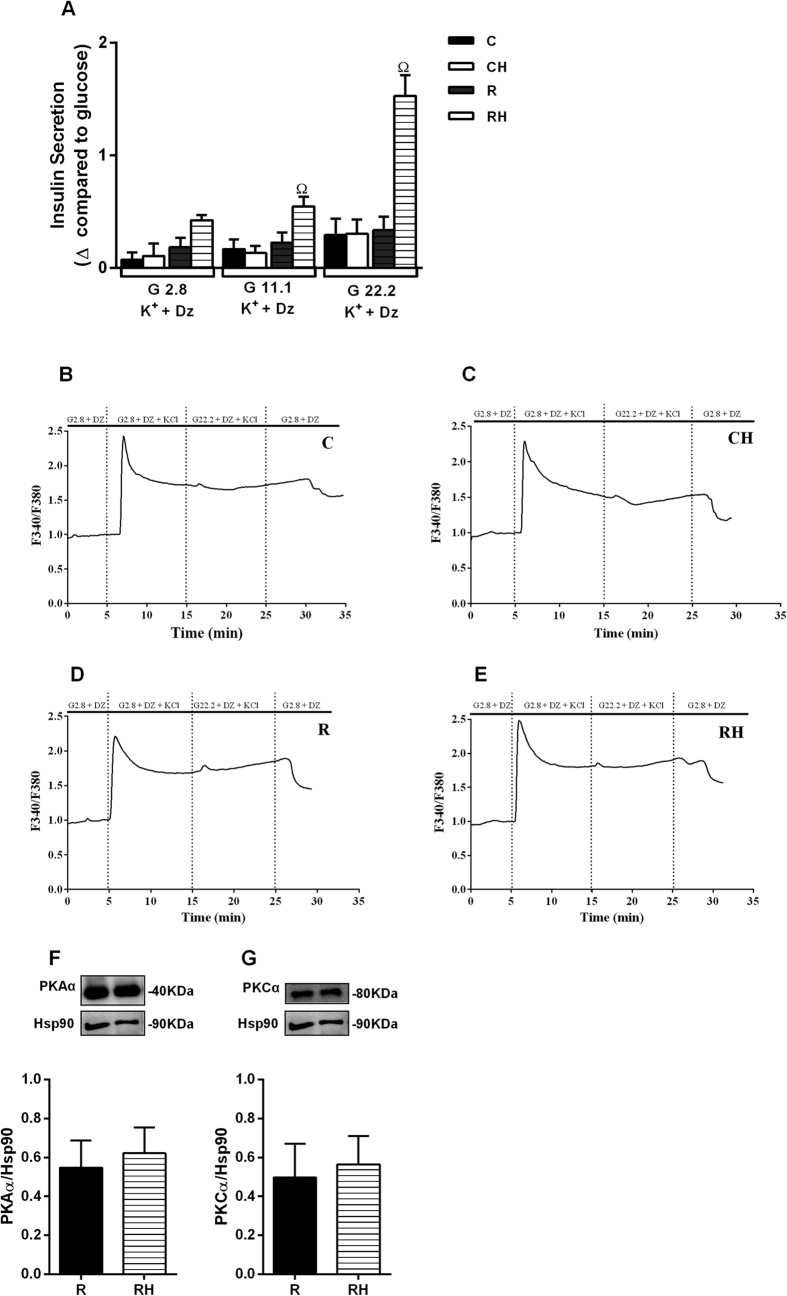
Islets from malnourished obese mice present increased contribution of amplifying pathway of insulin secretion: Insulin secretion in response to glucose 2.8; 11.1 or 22.2 mM in the presence of Dz (250 μM) and K^+^ (30 mM). Δ glucose value was calculated by subtraction of mean value of GSIS at respective glucose concentration plus K^+^ and Dz (**A**). Ca^2+^ influx induced by glucose (G2.8 or G22.2) in the presence of Dz and K^+^ in pancreatic islets (**B**–**E**). Protein content for PKAα (**F**), PKCα (**G**). The values are the ratio of F340/F380. The data are mean ± SEM from 3-4 independent experiments (n = 4–6 mice per group) (p < 0.05) ^Ω^R, in ANOVA Newman Keuls´s post-test.

The AMP PATH was studied while holding the K^+^_ATP_ channels open with diazoxide and depolarizing the islet cells with high concentrations of KCl in the presence of low or high glucose. In such experimental conditions, the increase in [Ca^2+^]i was similar in the presence of 2.8 and 22.2 mmol/L glucose (Fig. 2B–E), and the AMP PATH contribution was higher only in the islets from RH mice (Fig. 2A).

### Islets from malnourished obese mice present an altered mitochondrial profile and GDH interferes in the AMP PATH of insulin secretion

**Figure 3:**
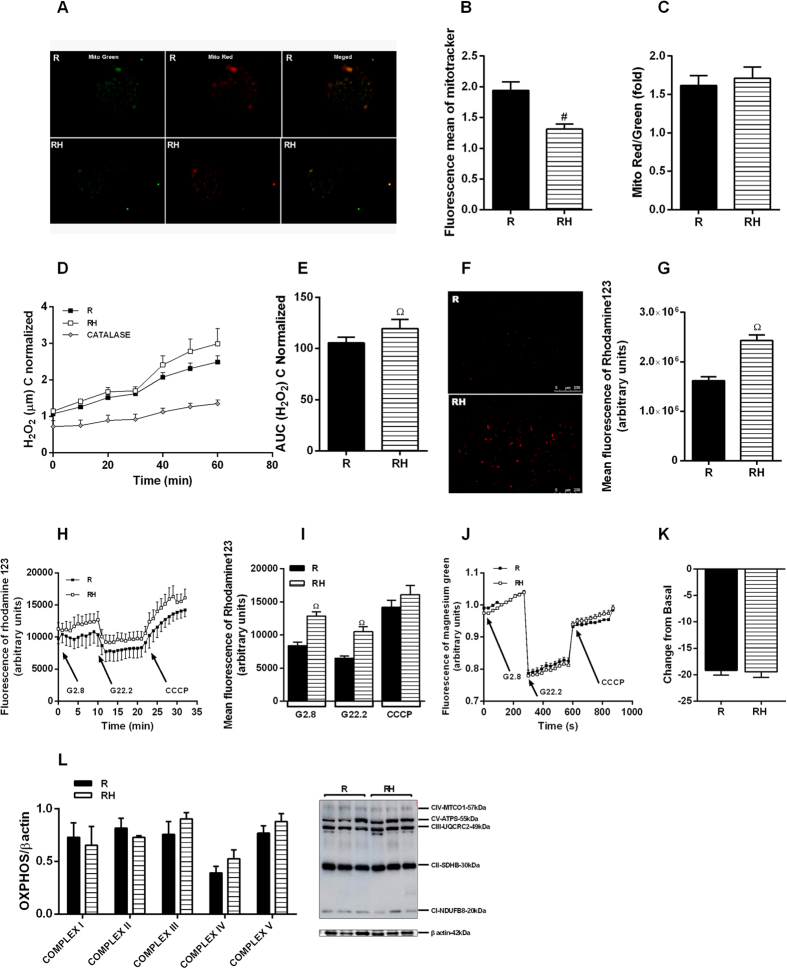
Islets from malnourished obese mice present increasing of mitochondrial profile: MitoTracker Red/Green staining of mitochondria in islets (**A**). Quantification of Mitotracker Red/Green ratio (**B**,**C**). H_2_O_2_ production (**D**,**E**). Indirect measure of mitochondria potential membrane from dissociated cells at baseline (**F**,**G**) or pancreatic islets incubated with rhodamine 123 in response to glucose (**H**,**I**). ATP flow showing by changes in magnesium green AM fluorescence in response to glucose (**J**,**K**). Protein content for OXPHOS (**L**). Results are means ± SEM (n = 4–9) (p < 0.05) ^Ω^R, in ANOVA Newman Keuls´s post-test.

PKAα and PKCα content did not change in the islets from RH mice (Fig. 2F,G). RH displayed decreased mitochondrial fluorescence compared to R (Fig. 3B). In addition, ATP flow was similar between R and RH (Fig. 3J,K). Despite it was not reported changes on the protein levels of the electron transport chain complexes (Fig. 3L), we observed a significant decrease in the mitochondrial membrane potential of RH islets (Fig. 3F–I), which was followed by increased levels of hydrogen peroxide (Fig. 3D).

**Figure 4:**
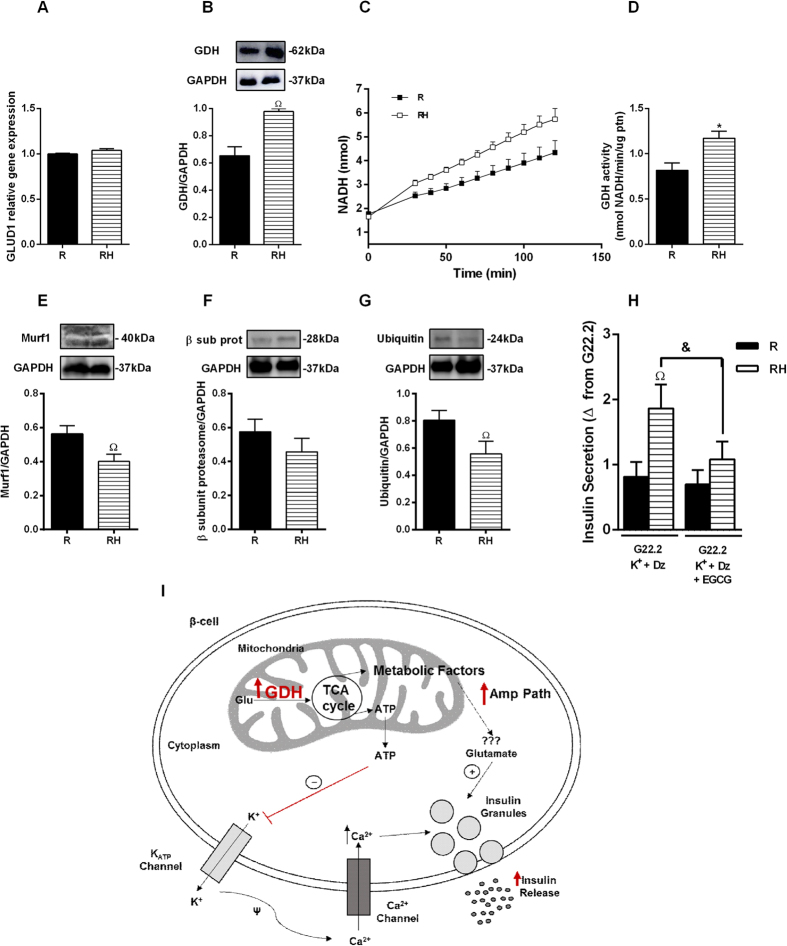
GDH modulates amplifying pathway of insulin secretion in islets from malnourished obese mice: mRNA expression for GLUD1/GAPDH (**A**). Protein content for GDH (**B**). NADH concentration (**C**). GDH activity (**D**) Pancreatic islets were isolated and incubated with glucose 22.2 mM in the presence of Dz (250 μM) and K^+^ (30 mM) with or without addition of epigallocatechin gallate (EGCG) (20 μM). The value Δ was obtained by subtracting of secretion with Glucose + K^+^ + Dz less insulin secretion with G22.2 (**E**). Protein content for murf 1 (**E**), proteasome β subunit (**F**) and ubiquitin (**G**). Overview of insulin secretion in β-cells mediated by GDH (**I**). Results are means ± SEM (n = 4–9) (p < 0.05) ^Ω^R, in ANOVA Newman Keuls´s post-test or ^&^as represented in Student t test.

Although no differences in GLUD1 gene expression were detected (Fig. 4A), GDH protein content and activity increased in the islets from RH (Fig. 4B–D). In agreement, the GSIS AMP PATH was almost inhibited in the presence of the GDH inhibitor, epigallocatechin-monogallate (EGCG) (Fig. 4H). RH group also displayed lower levels of ubiquitin proteasome pathway proteins, Murf1 and ubiquitin (Fig. 4E,G). GDH in CH did not change (Fig. S3G). The apoptosis levels were not different in the islets from the R, RH (Fig. S2A,B) and CH groups (Fig. S3F).

## Discussion

Protein malnutrition early in life leads to β cells dysfunction and a derangement of glucose metabolism during adulthood^11^. In addition, it worsens the deleterious effects of obesity on β cells, compromising the efficacy of therapeutic approaches to reestablish the triggering mechanisms of insulin secretion^18^. Insulin resistance alters the expression and/or activity of various proteins that form the K^+^_ATP_ and voltage dependent Ca^2+^ channels^21^,^22^. Because the triggering and AMP PATH work together to regulate insulin secretion^7^, the loss of one of these components may overload the other, leading to β cell failure; however, the impact of impaired triggering mechanisms on the AMP PATH of obese mice has not yet been addressed.

A higher fold change in body mass and the Lee index from the protein-restricted group shows a metabolic program outcome and might result from the increased energy intake supplied by the HFD, associated with lower energy expenditure. These results are in agreement with impaired hypothalamic insulin signaling previously reported in obese malnourished mice^23^.

Obesity reduced glucose tolerance and insulin sensitivity in normal- and low-protein-fed groups. In response to an increase in insulin resistance, insulinemia promptly increased in both groups. Thus, we proceeded to investigate pancreatic β cell function and observed that GSIS was also enhanced in the HFD-treated groups. Although the degree of insulin resistance was similar between CH and RH as judged by blood glucose uptake during the euglycemic-hyperinsulinemic clamp, the absolute levels of GSIS were lower in RH for a given glucose concentration, suggesting a possible signal of early pancreatic β cell failure.

While investigating the contribution of the AMP PATH to the enhanced GSIS in obese mice and the possible contribution brought by protein malnutrition, we observed that protein malnutrition (R) or HFD (CH) alone did not interfere in the AMP PATH; however, when mice were subjected to a low-protein diet followed by a HFD, an increased participation of the AMP PATH was detected. This outcome could be due to an acute response to a higher amplitude of intracellular Ca^2+^ influx^24^ in RH islets; however, no differences in the Ca^2+^ amplitude were reported in these groups^18^. Therefore, the increase in the AMP PATH might be a compensatory mechanism to maintain adequate levels of GSIS. Indeed, pancreatic islets and β cell lines chronically exposed to high levels of nutrients undergo changes in glucose metabolism, pyruvate shuttling, mitochondrial function and oxidative stress associated with altered GSIS^25^. In addition, islets from genetically obese (ob/ob) mice display higher firing frequency, intracellular Ca^2+^ mobilization, and oscillatory pattern, even at low glucose concentrations^26^,^27^. All of these changes point to compensatory adaptations to adequately adjust GSIS demand. Protein kinases A and C (PKAα and PKCα) activate the AMP PATH, potentiating insulin secretion^6^. In addition, GSIS amplification also depends on mitochondrial metabolism through the production of the so-called metabolic coupling factors. Thus, we attempted to clarify which of these components of the AMP PATH are modulated by protein malnutrition.

To establish which mechanisms are responsible for the increased response of the AMP PATH, we first analyzed PKAα and PKCα protein content. In contrast to other studies^28^,^29^, our model displayed no alterations in the content of these kinases; however, the mitochondrial profile was modified. Chronic exposure to high levels of nutrients increases β cell mitochondrial membrane potential, enhancing the triggering and AMP PATH in non-protein-restricted mice^26^,^30^. In our RH mice, the mitochondrial content and the inner membrane potential were reduced, even at non-stimulatory glucose concentrations. Mitochondrial protein expression and metabolism are altered in response to protein malnutrition or HFD^31^,^32^. A low-protein diet reduces the content of several mitochondrial proteins including respiratory chain proteins, leading to reduced ATP production^33^. Thus, protein restriction seems to compromise mitochondrial function elicited by a HFD in β cells from RH, leading to reduced membrane potential despite no differences reported on mitochondrial complexes content and ATP flow. These data suggest dysfunctional activity of mitochondrial complexes in obese malnourished mice, since the protein content was not altered. Considering that the mitochondrial inner membrane potential was reduced in RH, we investigated the shuttling cycles that have been proposed as the source of other metabolic coupling factors for insulin secretion. GDH has a close relationship with GSIS^34^,^35^,^36^. In addition, GDH was already reported to modulate the AMP PATH in lean mice, increasing glutamate production^37^. Thus, this enzyme may be involved with the increased participation of the AMP PATH on GSIS in obese malnourished mice. Whereas the expression of GDH mRNA was similar between the groups, the protein content and activity were increased in RH islets, correlating positively with the GSIS AMP PATH, indicating that GDH protein may undergo post-translational modulation. Considering the reduced amino acids supply during protein malnourishment, we hypothesized that cells would reduce protein degradation rather than increase protein synthesis. Thus, we measured proteins from the ubiquitin proteasome, and reported lower content in two of them in obese malnourished mice. These results led us to propose that protein degradation should be lower in RH, and this modulation might explain the higher protein content of GDH found in this group.

Indeed, the participation of GDH on the enhanced AMP PATH was confirmed when insulin secretion was restored in RH islets incubated in presence of Dz, KCl and EGCG. In agreement, higher mitochondrial shuttling cycles were reported in obese Zucker rats, providing evidence that obesity enhances the AMP PATH to support their increased GSIS^32^. These findings support that mitochondria are intracellular targets for malnutrition-induced β cell programming^15^. Mitochondrial dysfunction reported in RH (i.e., reduced membrane potential) is associated with higher ROS production^38^, which may trigger signaling pathways involved with β cell death^39^. In an attempt to establish a possible relationship between β cell dysfunction and death in this model, we measured ROS and cell viability. ROS content was increased in RH whereas in CH it was not altered; however, we did not detect evidence of increased apoptosis or cell death in any group, indicating that at this stage of treatment, there was only impairment of β cell function. Nevertheless, the protein malnutrition-induced earlier exposure to higher levels of ROS might reduce β cell viability and predispose its failure. In addition to the classical DOHaD concept, that establishes the origins of metabolic diseases in early life periods, our group and others have shown that the protein deprivation, during the adolescence also alters glucose homeostasis, showing a new window for the metabolic programing. Moreover, the association of protein malnutrition followed by a high fat diet impairs metabolic control, leading to glucose intolerance and insulin resistance in adulthood^18^,^40^,^41^. The increase in ROS production in RH islets provides evidence for the malnutrition-induced metabolic reprograming, favoring degenerative processes, such as oxidative stress, which might contribute to the reduced viability of β cells.

In conclusion, protein malnutrition programming of β cells induces mitochondrial dysfunction, changing the response to a HFD challenge. Whereas the signaling of the triggering pathway through ATP production is impaired, the AMP PATH is enhanced due to increased GDH. Further studies are needed to establish the mechanisms of malnutrition-induced mitochondrial impairment in response to nutrient overload and its role on β cell failure and T2D onset.

## Experimental Procedures

### Animals and dietary interventions

All animal experiments were carried out in accordance with the protocols approved by the Animal Care and Use Committee of the Campinas University (UNICAMP) (number: 3057-1). C57Bl/6 mice were obtained from UNICAMP and maintained at 22 ± 1 °C in a 12-h light–dark cycle. Thirty-day-old mice were assigned into the following groups: normal protein diet (14% protein) (Control: C) or protein-restricted diet (6% protein) (Restricted: R). After 6 weeks, some of the C and R mice were fed either a normal or high fat diet (HFD) (35% fat) for 8 weeks (CH and RH).

### Physiological Measurements

The Lee Index was calculated^42^. The fat pads were weighed. Plasma glucose and insulin were measured using a glucometer (Accu-Chek Performa) and by radioimmunoassay (RIA), respectively.

For indirect calorimetry, the mice were allowed an 8 h acclimation to the apparatus. The mice remained at rest during a light/dark period for 24 h. O_2_ and CO_2_ were measured by an Oxylet system (Pan Lab/Harvard Apparatus). The respiratory quotient (RQ) was calculated from these data. Locomotor activity analysis was performed in multitake cages LE 001 PH (Pan Lab/Harvard Apparatus).

### Intraperitoneal glucose test and hyperinsulinemic–euglycemic clamp

For the intraperitoneal glucose test (ipGTT), mice were fasted overnight (12 h). An intraperitoneal glucose load (2 g/kg) was administered, and blood glucose measurements recorded at 0, 15, 30, 60, and 120 min via tail snip using a handheld glucometer. A hyperinsulinemic–euglycemic clamp (120 min) was carried out with a prime continuous insulin infusion (30 mU kg^−1^. min^−1^). Blood glucose was measured at 5 min intervals, and glucose (5% wt/vol) was infused at a variable rate to maintain blood glucose at fasting levels.

### Islet isolation, GSIS, and participation of AMP PATH of insulin secretion

Islets were isolated by collagenase digestion of the pancreas. Islets were incubated for 30 min at 37 °C in Krebs–bicarbonate buffer (KBB). This medium was then replaced with fresh buffer, and the islets were incubated for 1 h with 2.8, 11.1 and 22.2 mmol/L glucose. To investigate the AMP PATH of insulin secretion, 30 mmol/L K^+^ and 0.25 mmol/L diazoxide (Dz) were added to the medium containing 2.8 or 22.2 mmol/L glucose. The insulin content of the medium was measured by RIA.

### Measurement of oscillations of cytoplasmic Ca^2+^

Pancreatic islets were incubated with fura-2 acetoxymethyl ester (5 μmol/L) for 1 h at 37 °C in KBB. The medium was replaced, and the islets were plated in a chamber at 37 °C on the stage of an inverted microscope (Nikon UK). The islets were then perfused with albumin-free KBB, which contained 2.8 mmol/L or 22.2 mmol/L glucose with 0.25 mmol/L Dz and 30 mmol/L K^+^, as indicated in the figures. A ratio image was acquired every 5 seconds with an inverted epifluorescence microscope (Nikon Eclipse TE200, Tokyo, Japan). The data were obtained using ImageMaster 3 software (Photon Technology International)^18^.

### Western blot

For Western blotting, 20 μg of the total protein for PKAα (#903; SCBT), PKCα (#8393; SCBT), cleaved caspase-3 (#9662; Cell Signaling), UCP2 (#77363; Abcam), GDH (#400051; US-Biological), Murf1 (#32920; SCBT), Proteasome subunit β type 2 (#22650; Abcam), Ubiquitin (#7780; Abcam) and the housekeeping proteins GAPDH (#3873; Cell Signaling Technology) and HSP90 (#101494; SCBT) were resolved using 10% SDS-PAGE and electroblotted onto nitrocellulose membranes. Detection was performed by enhanced chemiluminescence (Pierce). The band intensities were quantified using ImageJ software.

### RNA extraction and qPCR

Total RNA from pancreatic islets was isolated with an AllPrep DNA/RNA Mini Kit from QIAGEN (#80204) and quantified using a NanoDrop 2000 (Thermo Fisher Scientific). cDNA was prepared using 1 μg total RNA and MultiScribe reverse transcriptase (Applied Biosystems). Mouse GLUD-1 gene (forward, 59- CTTCCCAGCAGAGTCAGTGC -39; reverse, 59- GGAAAACGCCACACACCTAC -39) expression was measured, and GAPDH (forward, 59- CCTGCACCACCAACTGCTTA -39; reverse, 59-GCCCCACGGCCATCACGCCA -39) used as a housekeeping gene. Real-time PCR was conducted using the StepOne thermocycler (Applied Biosystems).

### Fluorescent staining of mitochondria

MitoTracker Green and Red CMXRos (100 nmol/L) was loaded into intact pancreatic islets at 37 °C in a humidified incubator for 20 min and observed using a FLoid Cell Image Station (Life Technologies).

### Measurements of ATP flow and mitochondrial membrane potential (Ψ)

Islets were loaded with MgG acetoxymethylester (5 mmol/L, Molecular Probes) for 60 min or Rhod-123 (10 μg/mL, Sigma) for 20 min at 37 °C in a humidified incubator and then were perfused with KBB containing varying concentrations of glucose (G2.8 and G22.2), and the fluorescence intensity was measured using a SpectraMax M3 (Molecular Devices).

### Hydrogen peroxide production (Amplex Ultra Red)

Islets were transferred to 96 wells culture plates containing 100 μl of KBB solution with G22.2 and Amplex Ultra Red (50 μM) for 60 min at 37 °C, fluorescence intensity was measured using a SpectraMax M3 (Molecular Devices). Catalase (300 U/L) was used as a negative control.

### Quantification of HO-PI fluorescence

The percentage of dead and viable cells was assessed by quantifying the fluorescence of the DNA-binding dyes PI and HO 33342 (Sigma-Aldrich), respectively. The islets were incubated with HO-PI for 20 minutes (5 mg/ml) and observed using a FLoid Cell Image Station (Life Technologies).

### Assay of GDH activity

GDH activity was determined using a specific kit (Abcam 102527). Measurement of GDH activity was conduct using the SpectraMax M3 plate reader (Molecular Devices).

### Statistical Analysis

The data are presented as the means ± SEM, and the differences were considered significant when p < 0.05. Comparisons were performed using 2-tailed unpaired Student’s t test or ANOVA followed with Newman Keuls correction when necessary.

## Additional Information

**How to cite this article**: Leite, N. C. *et al.* Protein malnutrition potentiates the amplifying pathway of insulin secretion in adult obese mice. *Sci. Rep.* **6**, 33464; doi: 10.1038/srep33464 (2016).

## Supplementary Material

Supplementary Information
