# Supplementary material for: Protein malnutrition potentiates the amplifying pathway of insulin secretion in adult obese mice
Source: Sci Rep. 2016 Sep 16;6:33464. doi: 10.1038/srep33464 (PMC5025848; doi:10.1038/srep33464)
Supplement: Supplementary Information [file srep33464-s1.doc]

**Protein malnutrition potentiates the amplifying pathway of insulin secretion in adult obese mice**

Nayara Carvalho Leite^1*^, Flávia de Paula^1^, Patrícia Cristine Borck^1^, Jean Franciesco Vettorazzi^1^, Renato Chaves Souto Branco^1^, Camila Lubaczeuski^1^, Claudio Cesar Zoppi^1^, Antonio Carlos Boschero^1^, Everardo Magalhães Carneiro^1^

^1^Department of Structural and Functional Biology, Institute of Biology, University of Campinas (UNICAMP), Campinas, SP, CEP: 13083-865, Brazil.

*nayaracleite@gmail.com

**SUPLEMENTAR FIGURES**

**Table S1. Biometric and Biochemical Parameters:**

|  | C | CH | R | RH |
| --- | --- | --- | --- | --- |
| Body Weight  (g) | 25.93±1.04 | 33.93±0.98* | 20.75±0.54* | 30.65±1.07^Ω^ |
| Retroperitoneal Fat Pad  (g/100g) | 0.3±0.04 | 1.35±0.17* | 0.41±0.07 | 1.11±0.08^Ω^ |
| Epididymal Fat Pad  (g/100g) | 0.92±0.14 | 2.72±0.32* | 1.0±0.12 | 2.21±0.18^Ω^ |
| Lee Index | 0.315±0.01 | 0.327±0.01* | 0.302±0.01* | 0.332±0.02^Ω^ |
| Fasting Insulin  (ng/mL) | 0.96±0.31 | 1.19±0.4* | 0.60±0.33* | 0.93±0.39^Ω^ |
| Fasting Glucose  (mg/dL) | 78.33±4.23 | 83.6±4.03 | 74.7±5.38 | 92.3±8.45 |

Data are the means ± SEM of 4-6 mice of each group. Symbols represent statistical difference (p< 0.05) * C; ^Ω^ R, in the ANOVA with post-test Newman Keuls.

**FIGURE S1**

**B**

**A**

**A**

**C**

**D**

**E**

**F**

**Figure S1. High Fat Diet promotes increase of weight gain and diminishes energy expenditure:** Weight gain (21-105 days of age) (A). The corresponding area under the curve (AUC) is shown in panel B. Panels C and E represent the record for 24 hours of locomotor activity and energy expenditure, respectively. The average locomotor activity and energy expenditure during the light and dark period is shown in panel D and F (n=4-6 mice per group). Symbols above the bars represent statistical difference (p<0.05) * C; ^Ω^ R; ^&^ Light *versus* Dark in Student's t test.

**FIGURE S2**

**A**

**B**

**
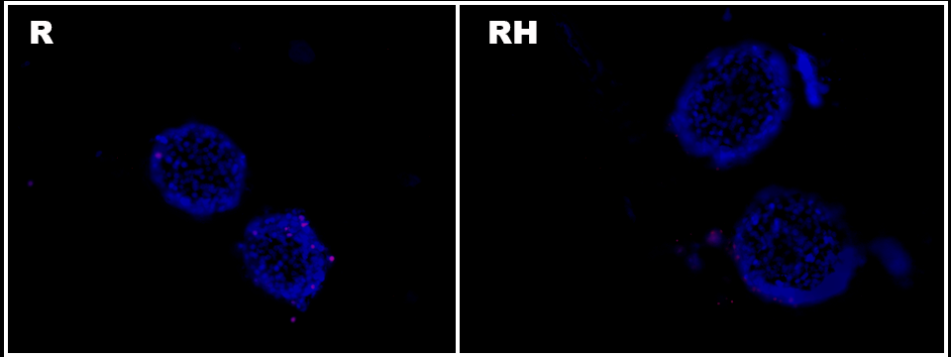
**


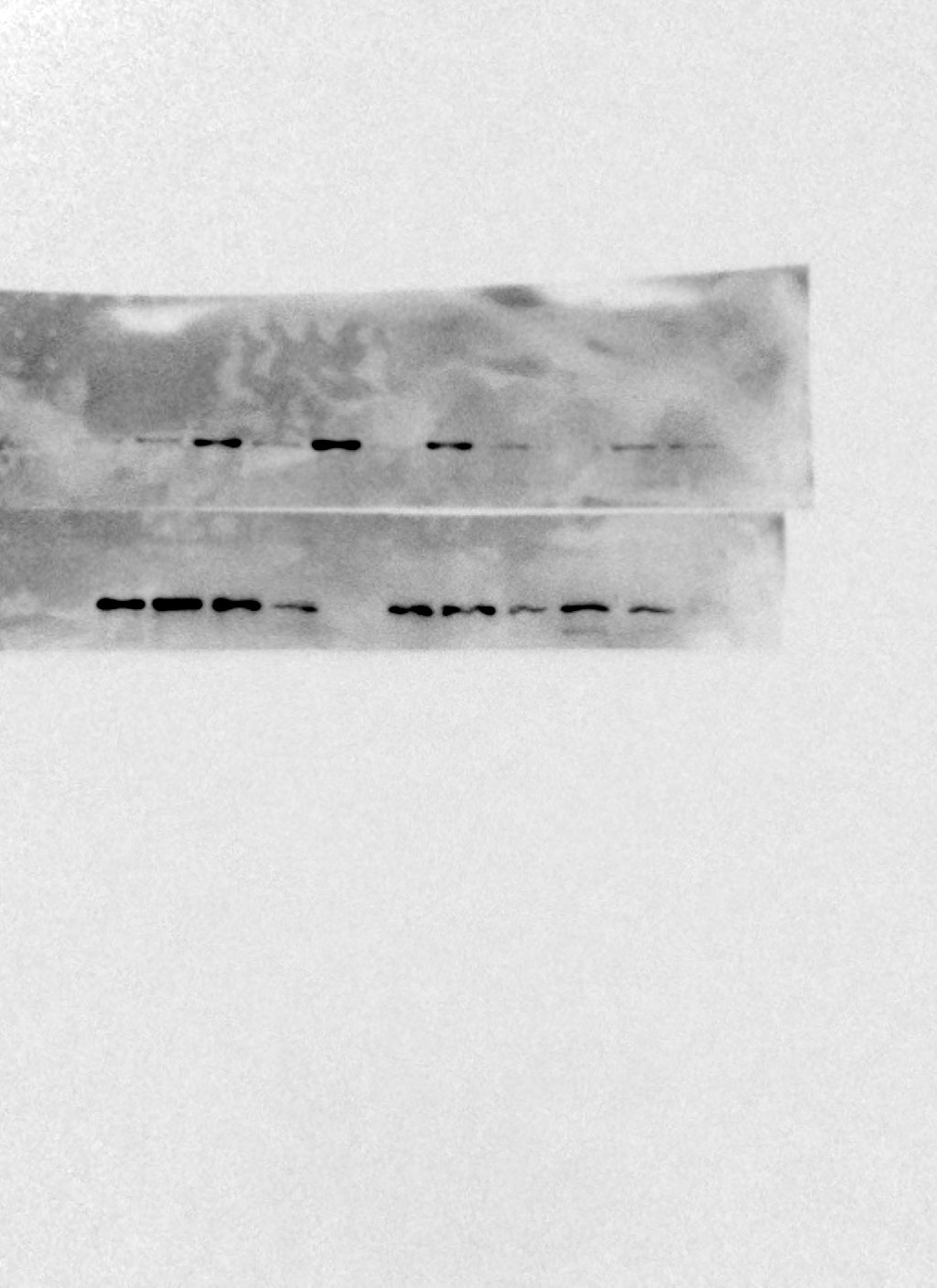


**-17kDa**

**-90 kDa**

**Cleaved**

**Caspase 3**

**HSP90**


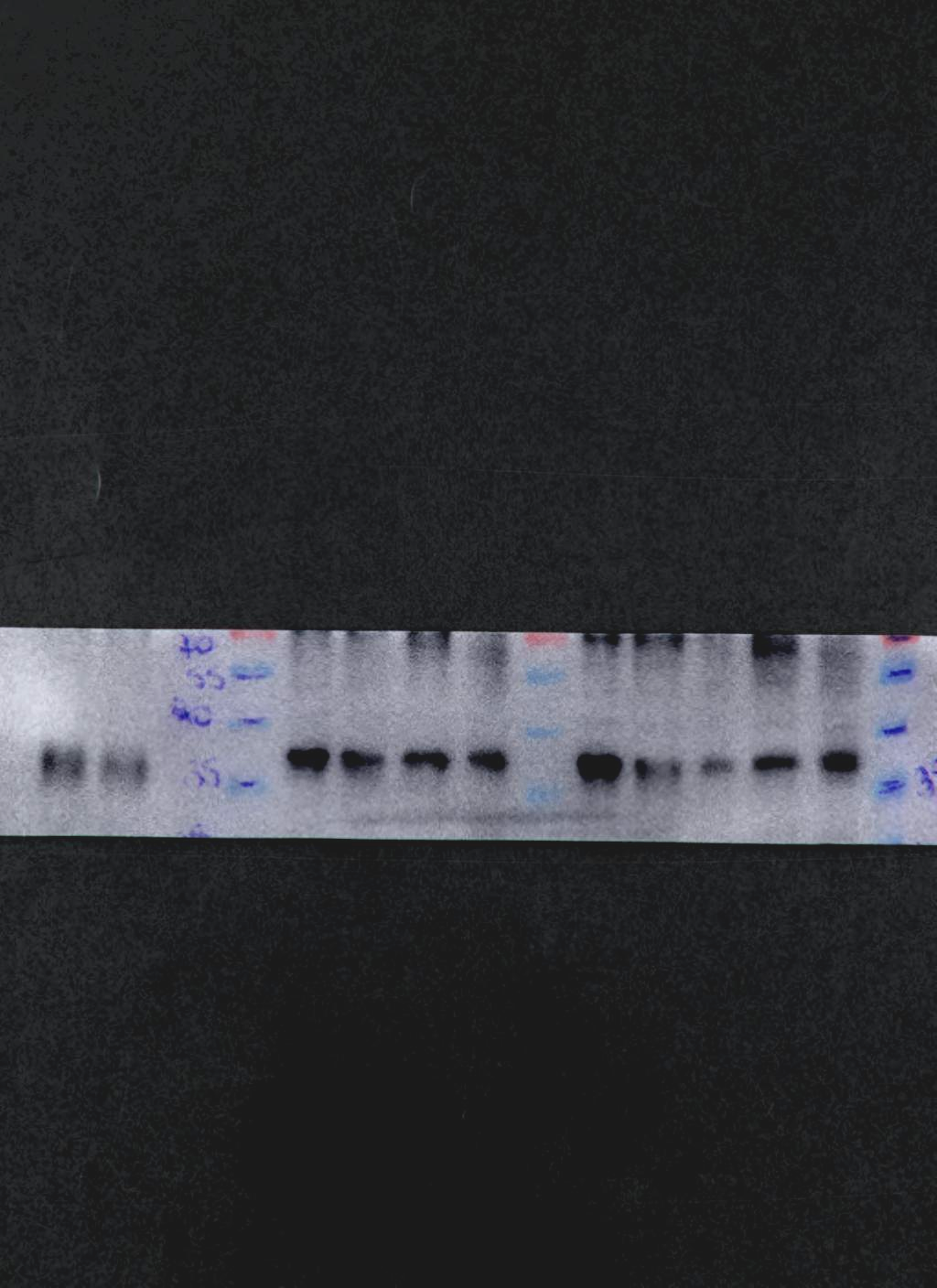


**Caspase 3**

**-32kDa**

**
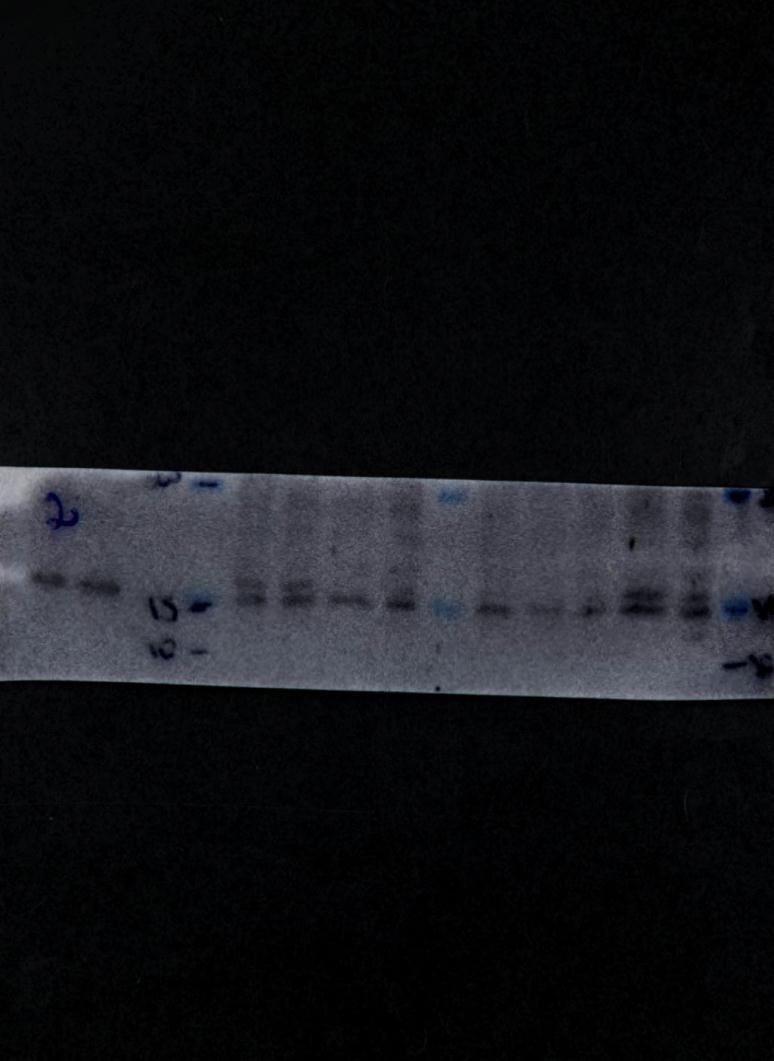
**

**Figure S2. Islets from malnourished obese mice do not present increase of apoptosis:** Representative images of isolated pancreatic islets stained for HO and PI (A). Protein content for cleaved caspase 3 (B). Results are means ± SEM (n= 4-9).

**FIGURE S3**

**A**

**C**

**
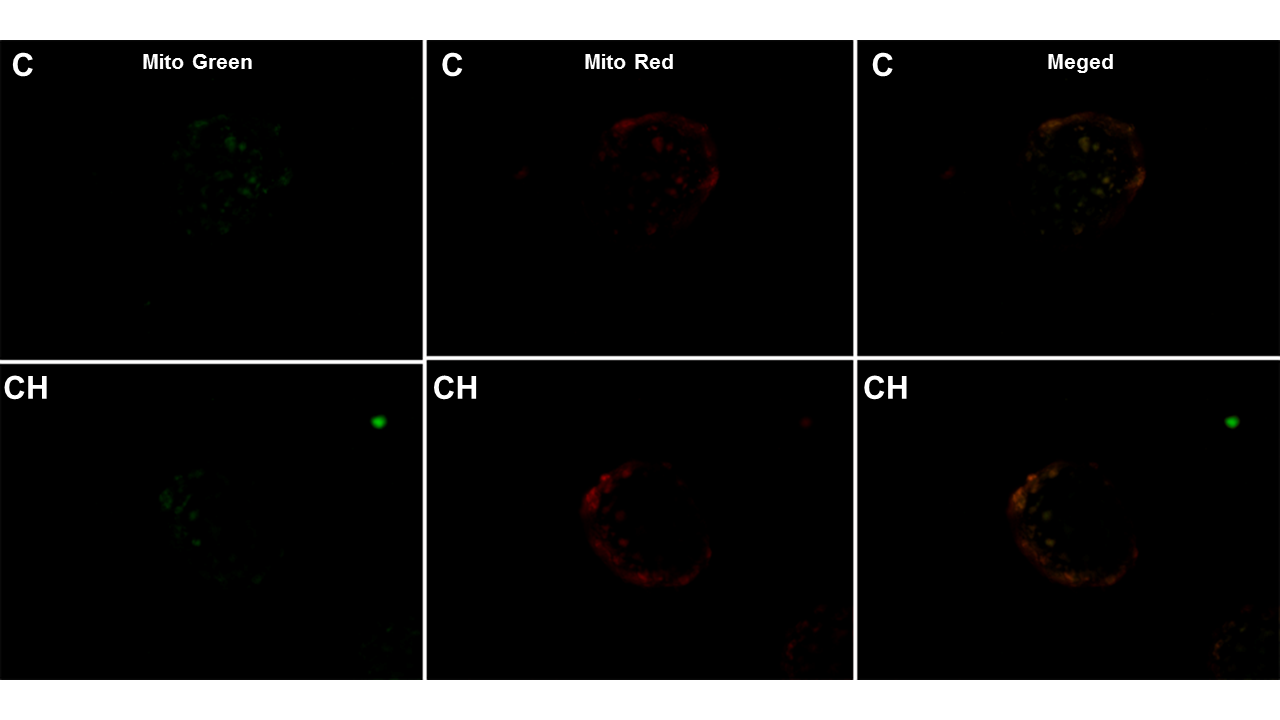
**

**B**

**B**

**B**

**F**

**D**


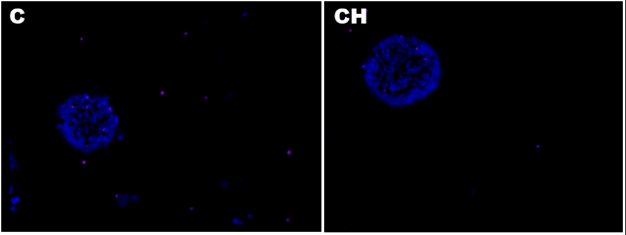

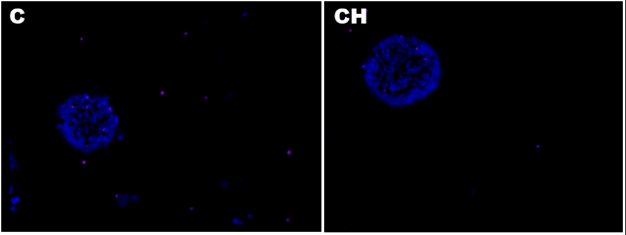


**E**

**H**

**G**

**I**

**J**


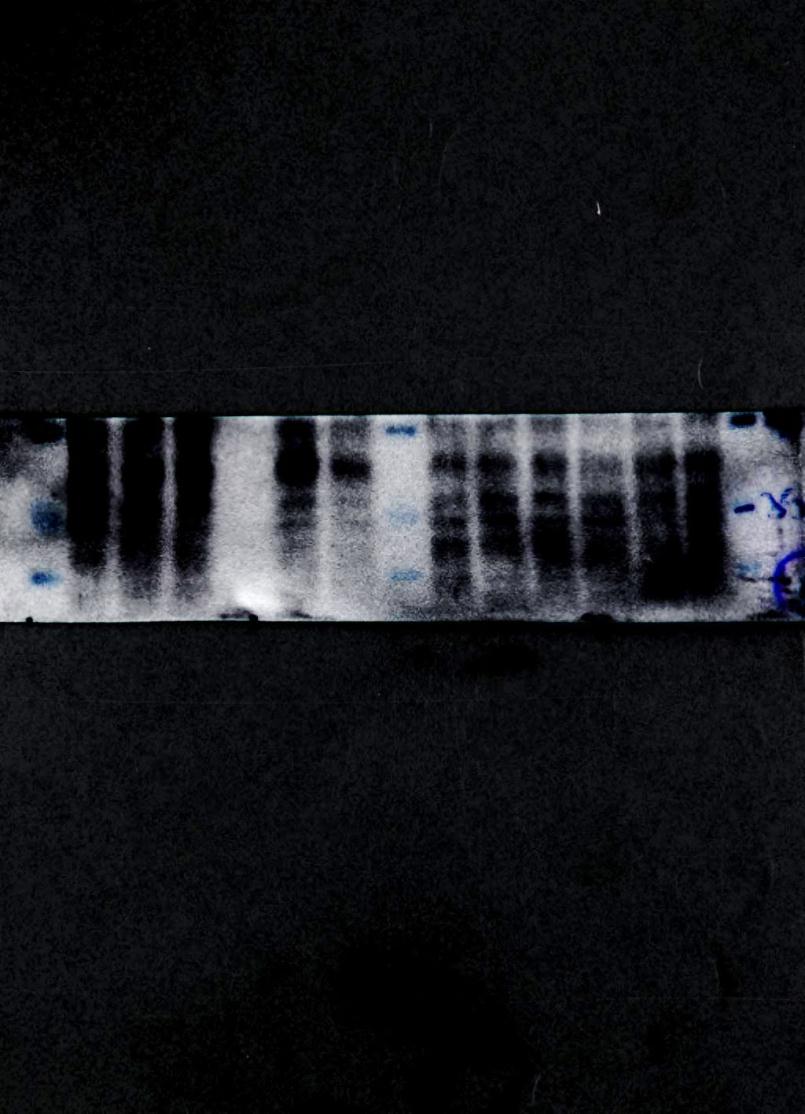

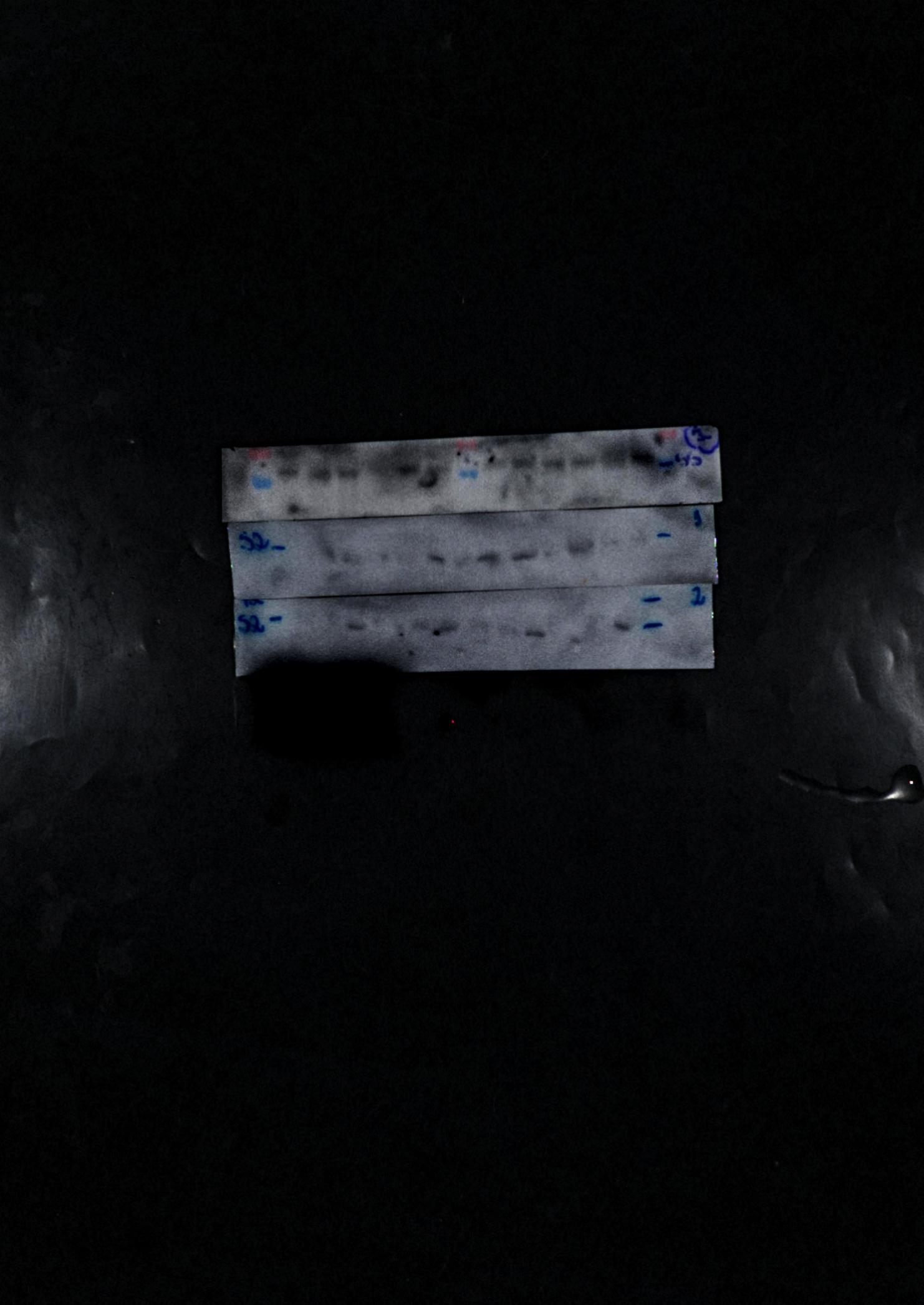


**GAPDH**

**GDH**

**-62KDa**

**-37KDa**

**Figure S3. Islets from obese mice does not present mitochondrial dysfunction neither increase of apoptosis and ubiquitination proteins:** MitoTracker Red/Green staining of mitochondria in islets (A). Quantification of Mitotracker Red/Green ratio (B,C). H_2_O_2_ production (D,E). Representative images of isolated pancreatic islets stained for HO and PI (F). Protein content for GDH (G), murf1 (H), proteasome β subunit (I) and ubiquitin (J). Results are means ± SEM (n= 4).
